# Diclofenac Prolongs Repolarization in Ventricular Muscle with Impaired Repolarization Reserve

**DOI:** 10.1371/journal.pone.0053255

**Published:** 2012-12-31

**Authors:** Attila Kristóf, Zoltán Husti, István Koncz, Zsófia Kohajda, Tamás Szél, Viktor Juhász, Péter Biliczki, Norbert Jost, István Baczkó, Julius Gy Papp, András Varró, László Virág

**Affiliations:** 1 Department of Pharmacology and Pharmacotherapy, University of Szeged, Szeged, Hungary; 2 Division of Cardiovascular Pharmacology, Hungarian Academy of Sciences, Szeged, Hungary; University of Milan, Italy

## Abstract

**Background:**

The aim of the present work was to characterize the electrophysiological effects of the non-steroidal anti-inflammatory drug diclofenac and to study the possible proarrhythmic potency of the drug in ventricular muscle.

**Methods:**

Ion currents were recorded using voltage clamp technique in canine single ventricular cells and action potentials were obtained from canine ventricular preparations using microelectrodes. The proarrhythmic potency of the drug was investigated in an anaesthetized rabbit proarrhythmia model.

**Results:**

Action potentials were slightly lengthened in ventricular muscle but were shortened in Purkinje fibers by diclofenac (20 µM). The maximum upstroke velocity was decreased in both preparations. Larger repolarization prolongation was observed when repolarization reserve was impaired by previous BaCl_2_ application. Diclofenac (3 mg/kg) did not prolong while dofetilide (25 µg/kg) significantly lengthened the QT_c_ interval in anaesthetized rabbits. The addition of diclofenac following reduction of repolarization reserve by dofetilide further prolonged QT_c_. Diclofenac alone did not induce Torsades de Pointes ventricular tachycardia (TdP) while TdP incidence following dofetilide was 20%. However, the combination of diclofenac and dofetilide significantly increased TdP incidence (62%). In single ventricular cells diclofenac (30 µM) decreased the amplitude of rapid (I_Kr_) and slow (I_Ks_) delayed rectifier currents thereby attenuating repolarization reserve. L-type calcium current (I_Ca_) was slightly diminished, but the transient outward (I_to_) and inward rectifier (I_K1_) potassium currents were not influenced.

**Conclusions:**

Diclofenac at therapeutic concentrations and even at high dose does not prolong repolarization markedly and does not increase the risk of arrhythmia *in normal heart*. However, high dose diclofenac treatment may lengthen repolarization and enhance proarrhythmic risk *in hearts with reduced repolarization reserve*.

## Introduction

Drug induced prolongation of cardiac repolarization that manifests on the surface ECG as QT interval lengthening is considered to represent enhanced risk for proarrhythmia and sudden cardiac death. Recently, several non-cardiac drugs were withdrawn from the market and the development of numerous compounds were halted at the preclinical stage due to their proarrhythmic effects, indicating that safety pharmacological concerns and monitoring in this respect are now more vigorous than they were in the past. Therefore, it can be speculated that several drugs approved earlier and used widely today would not reach clinical phase of development due to failing at current, more rigorous safety pharmacology tests. However, the vast majority of drugs developed earlier and successfully applied today do not enhance proarrhythmic risk in the normal heart. On the other hand, there are certain situations when cardiac repolarization reserve [1]–[3] is attenuated due to ion channel protein encoding gene mutations, diseases or even lifestyle when otherwise minor subthreshold drug effects on cardiac repolarization may enhance repolarization instability with subsequently elevated proarrhythmic risk.

As a possible example for such situations, a number of sudden deaths of young athletes were reported in the past several years. The majority of these events were sudden cardiac deaths (SCD) attributed to ventricular fibrillation. Fortunately, the incidence of SCD of young athletes is quite low (around 1–2:100 000), however, it is still 2–4 times more frequent than in the age-matched population not participating in competitive sport [4]. There are a number of factors that can play a significant role in the development of SCD in young athletes [5]. Cardiac hypertrophy is considered as one of the most important risk factors that has been associated with a characteristic electrical remodeling [6] that involves the downregulation of cardiac potassium channels, including I_Ks_, a repolarizing current that has a uniquely important role in cardiac ventricular repolarization reserve [7], [8]. Further important factors that influence arrhythmia disposition include various other cardiovascular diseases, hypokalemia, doping and seemingly harmless medications.

These medications may include non-steroid anti-inflammatory (NSAID) drugs often taken by athletes to alleviate pain related to sports injuries. Diclofenac is such a drug that is widely used for this purpose. Diclofenac intake has been associated with sudden death in four professional soccer players in a television show, however, scientifically this claim was not very well documented [9]. In this report, the pathologist Jørgen Lange Thomsen attributed these fatalities to coronary constriction as a possible consequence of cyclooxygenase enzyme (COX) inhibition [9]. However, one can speculate that different mechanisms may also be involved including direct cardiac electrophysiological effects on potassium channels with consequent changes in ventricular repolarization.

Diclofenac is a nonselective NSAID drug, which blocks COX-1 and COX-2 enzymes and is widely used as an anti-inflammatory and analgesic drug. There is growing concern regarding increased cardiovascular risks of NSAIDs application [10]–[12], however, very little is known about the cardiac electrophysiological effect of these drugs. Therefore, detailed characterization of the possible effects of diclofenac, one of the most frequently applied NSAIDs, on ventricular repolarization and transmembrane ionic currents and further consideration of the possible proarrhythmic potency of the drug seems reasonable since it can not be ruled out that its possible proarrhythmic potency might contribute to the higher incidence of SCD in young athletes. Such studies are justified by the fact that diclofenac is very often used in large doses for the treatment of sports injuries [13].

In the present study, the cellular electrophysiological effects of diclofenac were characterized including its effects on the main transmembrane ionic currents in single ventricular myocytes as well as on the action potential characteristics in canine isolated ventricular muscle and Purkinje fibers. The possible proarrhythmic potency of the drug was also investigated in an anaesthetized rabbit proarrhythmia model.

## Methods

### Ethics Statement

All experiments were carried out in compliance with the *Guide for the Care and Use of Laboratory Animals* (USA NIH publication NO 85–23, revised 1996) and conformed to the Directive 2010/63/EU of the European Parliament. The protocols have been approved by the Ethical Committee for the Protection of Animals in Research of the University of Szeged, Szeged, Hungary (approval number: I-74-5-2012) and by the Department of Animal Health and Food Control of the Ministry of Agriculture and Rural Development (authority approval number XIII/1211/2012).

### Conventional Microelectrode Technique

Adult mongrel dogs (8–14 kg) of either sex were used. Following sedation (xylazine, 1 mg/kg, i.v.) and anaesthesia (thiopental, 30 mg/kg i.v.), the heart was rapidly removed through right lateral thoracotomy. The hearts were immediately rinsed in oxygenated modified Locke’s solution containing (in mM): NaCl 120, KCl 4, CaCl_2_ 1.0, MgCl_2_ 1, NaHCO_3_ 22, and glucose 11. The pH of this solution was set between 7.35 and 7.4 when saturated with the mixture of 95% O_2_ and 5% CO_2_ at 37°C. Isolated muscle preparations obtained from the right ventricle and Purkinje fibers were individually mounted in a tissue chamber with a volume of 50 ml. These preparations were stimulated through a pair of platinum electrodes in contact with the preparation using rectangular current pulses of 2 ms duration. These stimuli were delivered at a constant cycle length of 1 s (500 ms for Purkinje fibers) for at least 60 min allowing the preparation to equilibrate before the measurements were initiated. Transmembrane potentials were recorded using conventional glass microelectrodes, filled with 3 M KCl and having tip resistances of 5–20 MΩ, connected to the input of a high impedance electrometer (Experimetria, type 309, Budapest, Hungary) which was coupled to a dual beam oscilloscope. The maximum diastolic potential, action potential amplitude, maximum upstroke velocity (V_max_) and action potential duration measured at 50% and 90% of repolarization (APD_50_ and APD_90_, respectively) were off-line determined using a home-made software running on an IBM compatible computer equipped with an ADA 3300 analogue-to-digital data acquisition board (Real Time Devices Inc., State Collage, PA, USA) having a maximum sampling frequency of 40 KHz. The following types of stimulation were applied in the course of the experiments: stimulation with a constant cycle length of 1000 ms (ventricular muscles); stimulation with a constant cycle length of 500 ms (Purkinje fibres); stimulation with different constant cycle lengths ranging from 300 to 5000 ms (or to 2000 ms in the case of Purkinje fibers to prevent spontaneous diastolic depolarization at cycle lengths longer than 2000 ms). Attempts were made to maintain the same impalement throughout each experiment. In case an impalement became dislodged, adjustment was attempted, and if the action potential characteristics of the re-established impalement deviated by less than 5% from the previous measurement, the experiment continued.

### Whole Cell Patch-clamp

Ventricular myocytes were enzymatically dissociated from dog hearts using the segment perfusion technique as described earlier in detail [7]. One drop of cell suspension was placed in a transparent recording chamber mounted on the stage of an inverted microscope. The myocytes were allowed to settle and adhere to the bottom for at least 5 minutes before superfusion was initiated. Only rod shaped cells with clear cross-striations were used. Cells were superfused with HEPES-buffered Tyrode solution containing (in mM): NaCl 144, NaH_2_PO_4_ 0.4, KCl 4.0, CaCl_2_ 1.8, MgSO_4_ 0.53, glucose 5.5, and HEPES 5.0. The pH was set to 7.4 and the temperature to 37^o^C.

Patch-clamp micropipettes were fabricated from borosilicate glass capillaries (Harvard Apparatus Ltd, Edenbridge, Kent, UK) using a micropipette puller (Flaming/Brown, type P-97, Sutter Co, Novato, CA, USA). These electrodes had resistances between 1.5 and 2.5 MΩ when filled with pipette solution containing (in mM): K-aspartate 100, KCl 40, ATP 5, MgCl_2_ 5, EGTA 4, CaCl_2_ 1.5 and HEPES 10. The pH of this solution was adjusted to 7.2 by KOH. When measuring potassium currents, 1 µM nisoldipine (gift from Bayer AG, Leverkusen, Germany) was added to the external solution to eliminate L-type Ca^2+^ current (I_Ca_). The slow component of the delayed rectifier potassium current (I_Ks_) was inhibited by using the selective I_Ks_ blocker HMR 1556 (0.5 µM). In some experiments, the rapid component of the delayed rectifier potassium current (I_Kr_) was blocked by 0.1 µM dofetilide. The L-type calcium current was recorded in HEPES buffered Tyrode’s solution containing 3 mM 4-aminopyridine in order to block the transient outward potassium current (I_to_) and a special K^+^ free pipette solution was used (composition in mM: CsOH 100, CsCl 20, TEACl 20, MgATP 5, HEPES 10, EGTA 4, CaCl_2_ 1.5, GTP 0.1, the pH was adjusted to 7.2 with aspartic acid). Membrane currents were recorded with Axopatch 200B patch-clamp amplifiers (Molecular Devices Inc., Sunnyvale, CA, USA) using the whole-cell configuration of the patch-clamp technique. After establishing a high resistance (1–10 GΩ) seal by gentle suction, the cell membrane beneath the tip of the electrode was disrupted by suction or application of short electrical pulses. The series resistance typically ranged from 4 to 8 MΩ before compensation (50%–80%). Experiments were discarded, when the series resistance was high or substantially increased during the measurement. Membrane currents were digitized after low-pass filtering at 1 kHz using analog-to-digital converters (Digidata 1322A and 1440A, Molecular Devices Inc., Sunnyvale, CA, USA) under software control (pClamp 8 and 10, Molecular Devices Inc., Sunnyvale, CA, USA). The same software was used for off-line analysis.

### ECG Measurements in Anaesthetized Rabbits

Male New Zealand white rabbits (2–3 kg) were used for the experiments. The animals were anaesthetized with thiopental (50 mg/kg, i.v.) given into the marginal vein of the right ear. A plastic catheter filled with isotonic saline containing 500 IU/mL heparin was inserted into the left carotid artery for the measurement of arterial blood pressure. The right jugular vein was cannulated for i.v. drug administration. The animals were allowed to stabilize for 20 min before baseline measurements were taken.

The blood pressure and the electrocardiogram (limb leads I, II and III) were continuously recorded (at 2 kHz), digitized and stored on a computer for off-line analysis using National Instruments data acquisition hardware (National Instruments, Austin, TX, USA) and SPEL Advanced Haemosys software (v3.2, Experimetria Ltd., Budapest, Hungary). The RR and QT intervals were measured as the average of 10 beats. During the measurement of the QT interval in anaesthetized rabbits, the guidelines described by Farkas *et al*. [14] were followed. The frequency corrected QT interval (QTc) was calculated by a formula specifically worked out for anaesthetized rabbits by Batey and Coker [15] for more accurate monitoring of heart rate dependent changes in the QT interval, as follows: QTc = QT – (0.704 * (RR-250)).

All intravenous infusions were administered using a programmable infusion pump (Terufusion TE-3, Terumo Europe, Leuven, Belgium). The first group of rabbits received 3 mg/kg diclofenac (Sigma-Aldrich, Hungary) in a 10 min infusion in a volume of 2 ml/kg followed by 25 µg/kg dofetilide (Gedeon Richter Ltd., Budapest, Hungary) with 15 min equilibrium between infusions. The second group was administered the same dose of compounds in the reverse order.

### Statistics

The incidence of TdP was compared using the χ^2^–test, with Yates’ correction. All other data are expressed as arithmetic mean ± S.E.M. values. Statistical differences were evaluated with one-way analysis of variance (ANOVA). Differences were considered significant when *p*<0.05.

## Results

### Effects of Diclofenac on Action Potential

**Figure 1:**
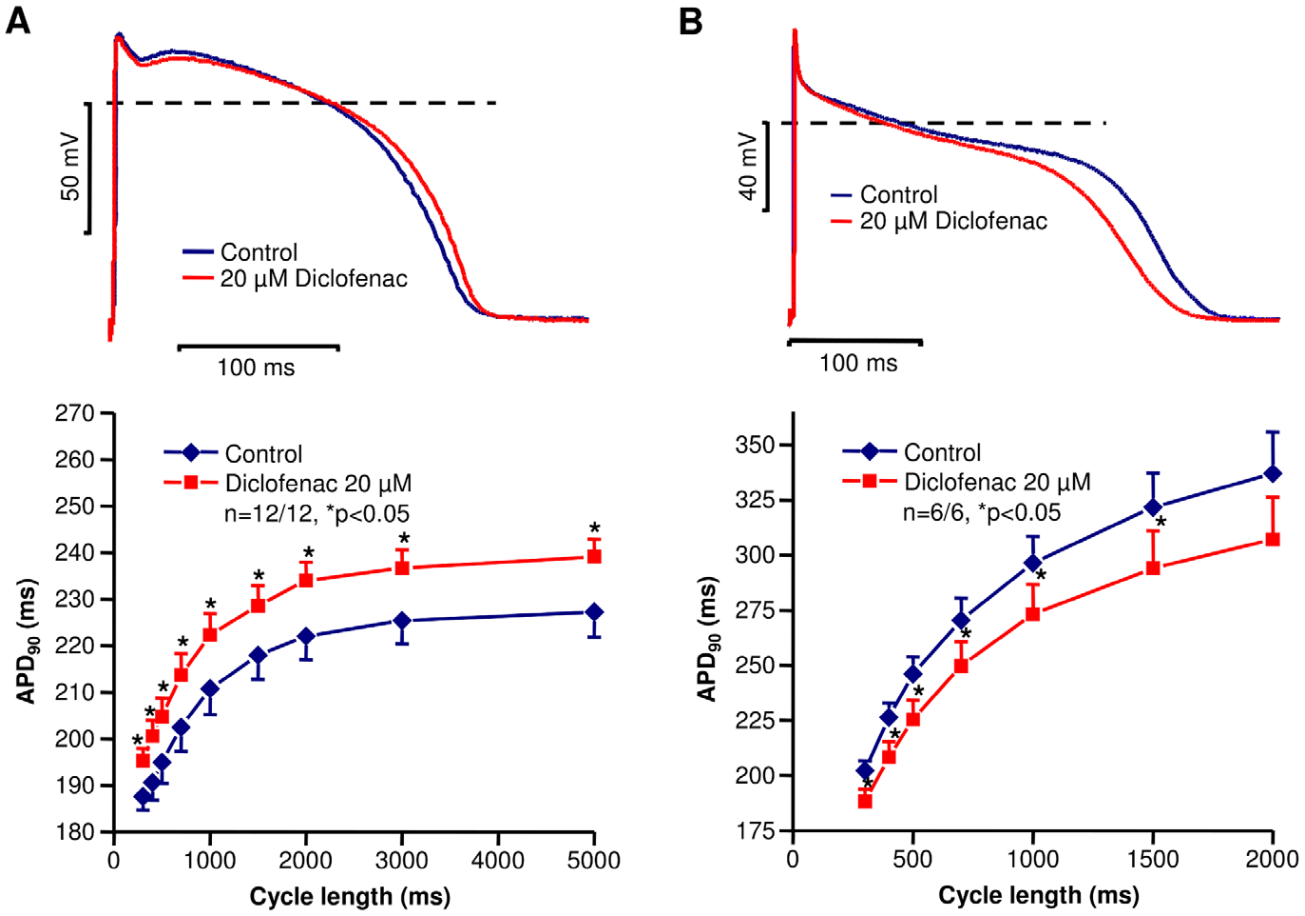
Effect of diclofenac on action potentials in canine right ventricular muscle preparations and in Purkinje fibers. Representative superimposed records (*top*) demonstrating the effect of 20 µM diclofenac on action potential configuration at 1 s stimulation cycle length (**A**, right ventricular muscle; **B**, Purkinje fiber). Cycle length dependent changes in action potential duration (APD_90_) measured under control conditions and in the presence of 20 µM diclofenac (*bottom*) in canine right ventricular muscle preparations (**A**) and in Purkinje fibers (**B**). Data are expressed as mean ± SEM, n = number of measurements/number of animals.

The effects of diclofenac on action potential configuration were studied in canine right ventricular muscle preparations and Purkinje fibers. The result obtained is shown in Figure 1A top panel. Small, but statistically significant action potential lengthening was induced by diclofenac (20 µM) at a basic stimulation frequency of 1 Hz in right ventricular muscle preparations (APD_90_ from 222.3±4.1 ms to 232.2±3.4 ms; APD_50_ from 185.3±5.7 ms to 198.3±4.1 ms; n = 13/13 animals, p<0.05). The maximum upstroke velocity was also decreased by the drug (control: 168.8±15.7 V/s, 20 µM diclofenac: 136.6±13.2 V/s, n = 13/13 animals, p<0.05) at cycle length of 1000 ms. To study the rate-dependent effect of the drug on APD_90_, the preparations were stimulated at cycle lengths ranging from 300 to 5000 ms. Under these circumstances diclofenac produced a slight rate-independent APD prolongation (Figure 1A bottom panel). In canine Purkinje fibers, however, the drug significantly shortened the action potential duration (APD_90_ from 248.1±10.9 ms to 230.8±9.7 ms; APD_50_ from 177.8±12.8 ms to 152.0±12.5 ms; n = 6 preparations/6 animals, p<0.05) and decreased V_max_ (from 673.9±8.5 V/s, to 562.4±27.0 V/s, n = 6 preparations/6 animals, p<0.05) at basic cycle length of 500 ms indicating a sodium channel blocking property of the drug [16]. The shortening of APD_90_ was rate-independent (Figure 1B).

**Figure 2:**
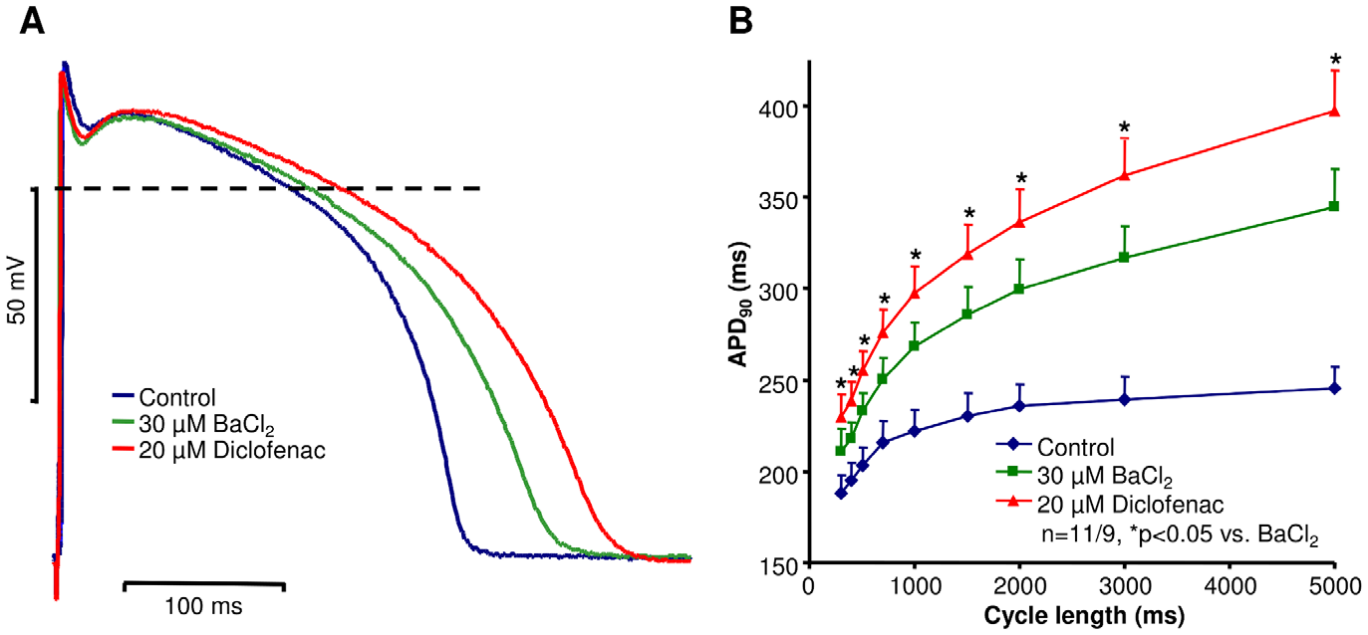
Effect of diclofenac on action potential repolarization in canine right ventricular preparations with impaired repolarization reserve. (A) Representative superimposed action potentials recorded from canine right ventricular muscle preparation at cycle length of 1 s. In these experiments 30 µM BaCl_2_ was applied to attenuate the repolarization reserve prior to 20 µM diclofenac superfusion. (B) Cycle length dependent changes in APD_90_ measured under the specified experimental conditions in canine right ventricular muscle preparation. Data are expressed as mean ± SEM, n = number of measurements/number of animals.

The influence of diclofenac on action potential repolarization in preparations with impaired repolarization reserve was also investigated. Repolarization reserve was greatly attenuated by the application of 30 µM BaCl_2_, which partially blocks the inward rectifier potassium current (I_K1_) in dog right ventricle [17]. BaCl_2_ lengthened APD in a reverse frequency dependent manner (Figure 2). In the presence of BaCl_2_, 20 µM diclofenac was added to these preparations. The drug induced a marked further lengthening relative to the APD_90_ values measured after the administration of BaCl_2_ (APD_90_: diclofenac: 309.8±15.2 ms vs. BaCl_2_: 283.5±15.3 ms; APD_50_: 241.0±10.3 ms vs. 225.2±12.6 ms; n = 11 preparations/9 animals, p<0.05, at cycle length of 1000 ms), i.e. APD lengthening effect of diclofenac was significantly augmented in preparations where the “repolarization reserve” was attenuated by previous application of BaCl_2_ (Figure 2). Under these circumstances the drug produced reverse rate-dependent APD prolongation (Figure 2B).

### Rationale for the Use of Anaesthetized Rabbit Proarrhythmia Model

Since the introduction of the Carlsson’s rabbit proarrhythmia model [18], the anaesthetized rabbit has been extensively used in different *in vivo* proarrhythmia studies for the assessement of arrhythmia risk associated with a compound of interest (for a comprehensive review see [19]). Therefore, two sets of experiments were also carried out in anaesthetized rabbits investigating the effects of diclofenac alone and in combination with the I_Kr_ blocker dofetilide (impairing repolarization reserve) on cardiac repolarization and the development of Torsades de Pointes ventricular tachycardia (TdP).

### Effect of Diclofenac, Dofetilide and their Combinations on QTc, RR Intervals and Incidence of TdP in Anaesthetized Rabbits

**Figure 3:**
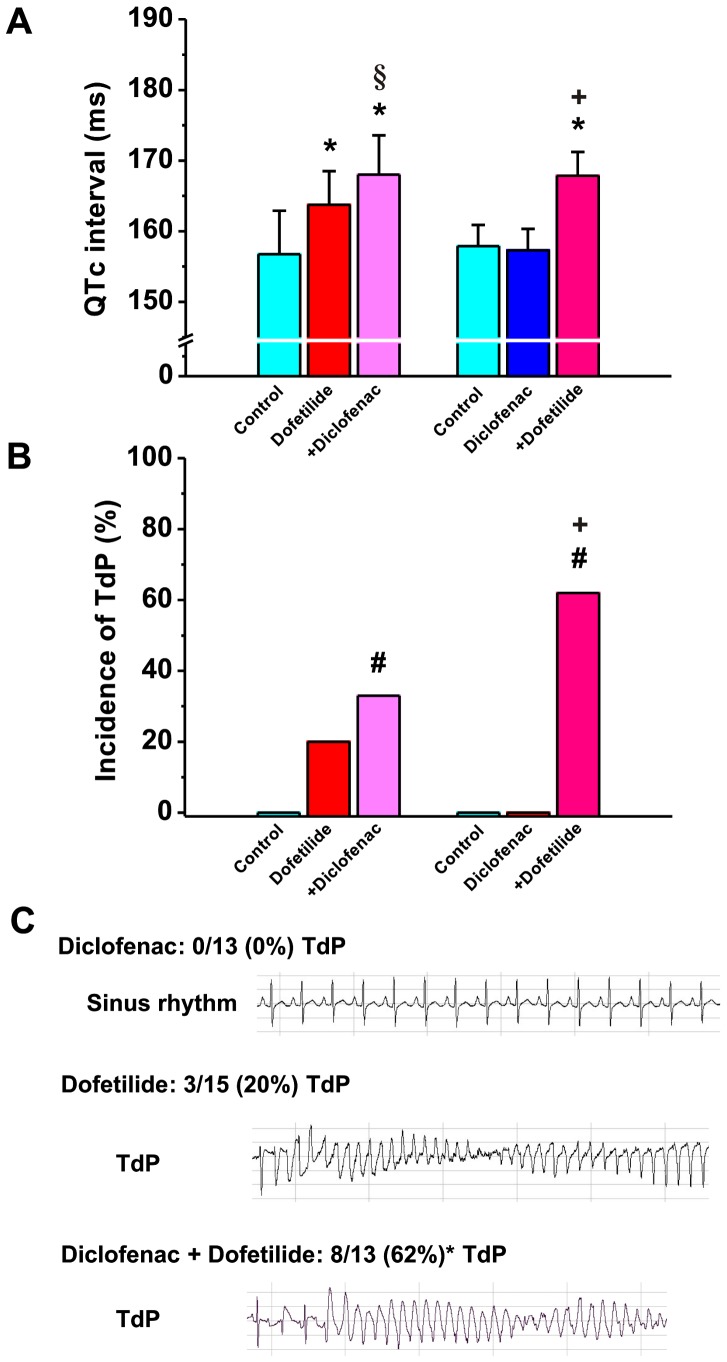
Effect of diclofenac on repolarization and TdP incidence in anesthetized rabbits. (**A**) Frequency corrected QT intervals (QTc) and (**B**) incidence of Torsades de Pointes ventricular tachycardia (TdP) in anaesthetized rabbits before and following dofetilide (25 µg/kg), dofetilide+diclofenac (3 mg/kg) and diclofenac, diclofenac+dofetilide administration. *p<0.05 vs. control, ^+^p<0.05 vs. diclofenac, ^§^p<0.05 vs. dofetilide, n = 15 and 13 animals/group, respectively. (**C**) Representative ECG recordings illustrate TdP development only after dofetilide or diclofenac+dofetilide combination, but not following diclofenac administration. #p<0.05 vs. baseline, n = 13 and 15 animals/group, respectively.

The QT and RR intervals were not different in baseline conditions between the two groups of anaesthetized rabbits, and were 147.6±4.95 vs. 144.9±7.18 ms and 242.8±4.28 vs. 229.4±6.02****ms, n = 15 and 13 animals, respectively, all *p*>0.05. Diclofenac (3 mg/kg) did not increase the RR interval either when given first (230.2±6.98 vs. 229.4±6.02 ms in control, *p*>0.05) or when it was administered following dofetilide infusion (252.1±7.73 ms vs 250.4±5.97 ms, *p*>0.05). Dofetilide, on the other hand, increased the RR interval both when given first (250.4±5.97 ms vs. 242.8±4.28 ms, *p*<0.05) and when it was administered following diclofenac infusion (240.3±8.21 ms vs. 230.2±6.98 ms, *p*<0.01). As shown on Figure 3A, diclofenac (3 mg/kg) did not change the QTc interval, while as expected, dofetilide (25 µg/kg) significantly lengthened QTc in anaesthetized rabbits. The combination of diclofenac and dofetilide significantly prolonged QTc, irrespective of the order of administration (Fig. 3A). Figure 3B and C illustrate the incidence of Torsades de Pointes arrhythmias in anaesthetized rabbits following the administration of diclofenac, dofetilide and their combination. Importantly, diclofenac alone did not induce TdP in any of the 13 animals examined, while TdP incidence following dofetilide was 20%, which corresponds well with our previous observations in this model [20]. However, the animals were not devoid of arrhythmias after diclofenac administration: 8 out of 13 exhibited frequent ventricular extra beats, and 7 out of 13 developed bigeminy. On the other hand, the combination of diclofenac and dofetilide led to a significant increase in the incidence of TdP and the increase was highest (62%) when diclofenac was administered first, indicating that further potassium channel inhibition following prior impairment of repolarization reserve can lead to increased frequency of serious arrhythmia development (Fig. 3B).

### Effects of Diclofenac on Transmembrane Ionic Currents

**Figure 4:**
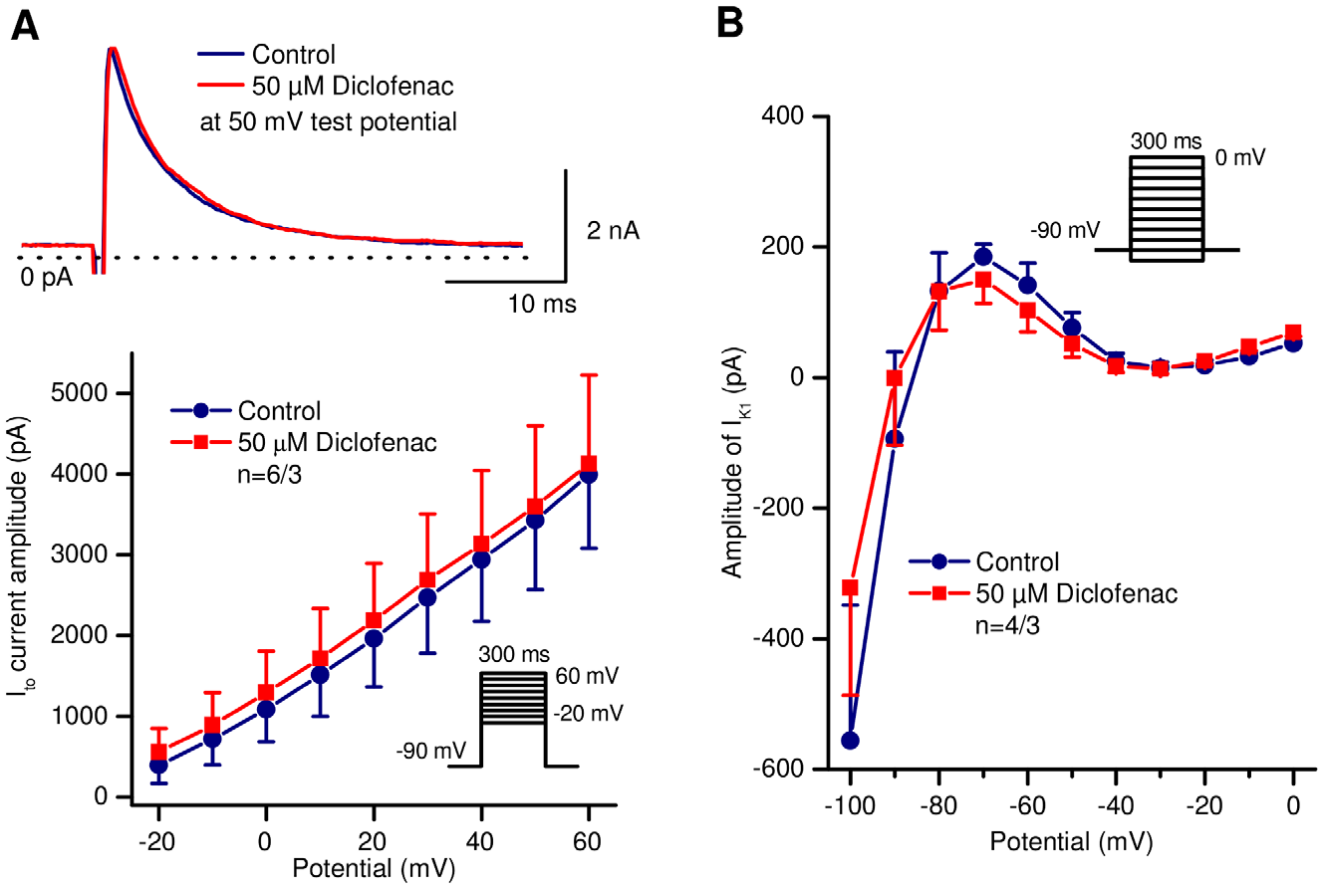
Lack of effect of diclofenac on the transient outward potassium (I_to_) and on the inward rectifier potassium (I_K1_) currents in canine ventricular myocytes. A, *top*: Representative I_to_ current traces under control conditions and after application of 50 µM diclofenac. A, *bottom*: Current – voltage relationships of I_to_ under control conditions and in the presence of 50 µM diclofenac. Panel B shows steady-state current – voltage relationships of I_K1_ before and after application of 50 µM diclofenac. Insets depict the voltage protocol applied during measurements. Data are expressed as mean ± SEM, n = number of measurements/number of animals.

The effects of the drug on the 4-aminopyridine sensitive I_to_, as well as on I_K1_, I_Kr_, I_Ks_ and I_Ca_ were investigated in canine ventricular myocytes. As shown in Figure 4A and 4B, diclofenac (even at 50 µM concentration) did not influence I_to_ or I_K1_ currents. I_to_ current was activated by 300 ms long depolarizing voltage pulses from the holding potential of −90 mV to test potentials ranging from −20 to +60 mV with a pulse frequency of 0.33 Hz. The amplitude of I_to_ was measured as the difference between the peak and the sustained current at the end of the voltage pulse. I_K1_ current was measured as the steady-state current level at the end of the 300 ms long voltage pulse in the voltage range of −100 to 0 mV with a pulse frequency of 0.33 Hz. The holding potential was −90 mV.

**Figure 5:**
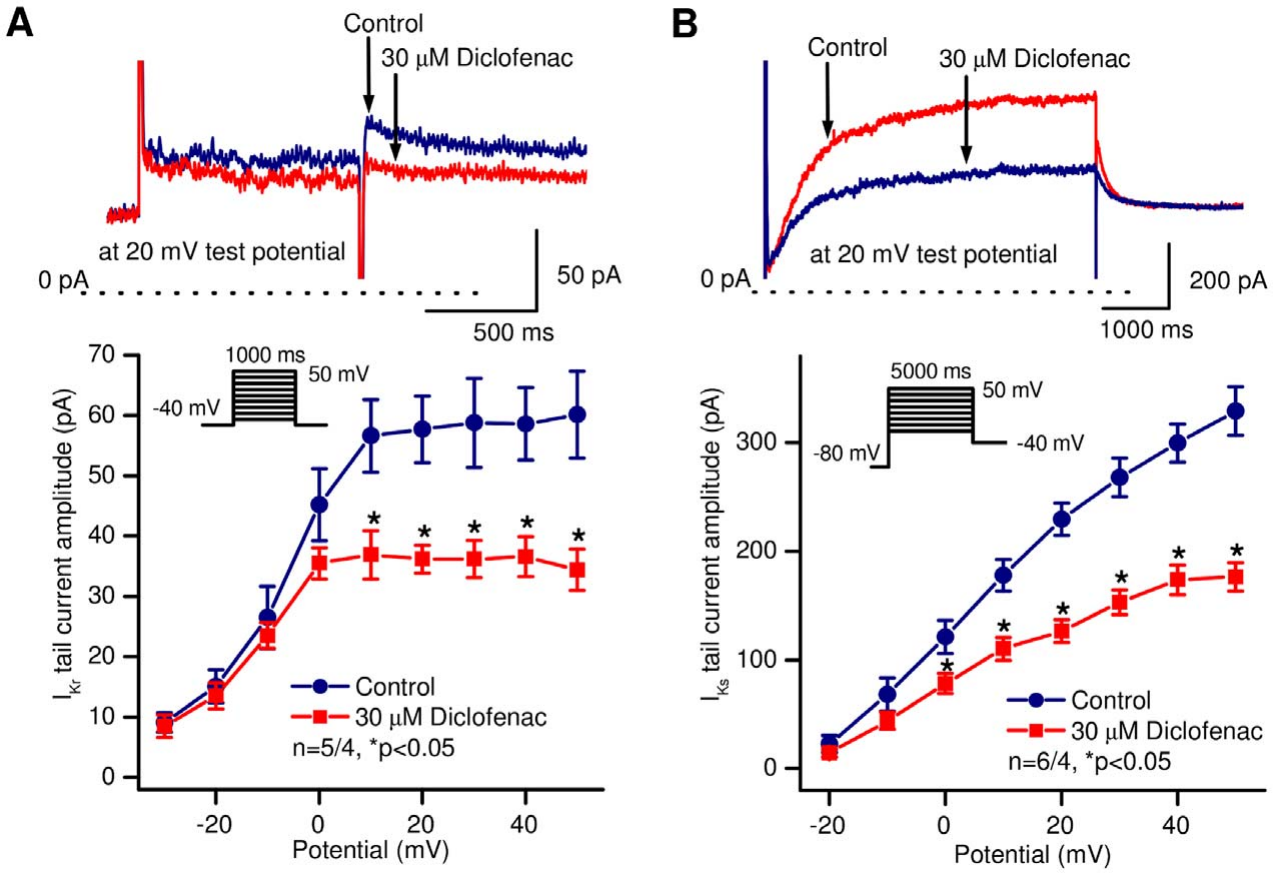
Effect of diclofenac on the rapid (I_Kr_) and slow (I_Ks_) component of the delayed rectifier potassium currents in canine ventricular myocytes. *Top* panels show representative current traces (A, I_Kr_; B, I_Ks_), *bottom* panels represent current – voltage relationships under control conditions and in the presence of 30 µM diclofenac. Insets indicate the voltage protocol applied during measurements. Data are expressed as mean ± SEM, n = number of measurements/number of animals.

I_Kr_ and I_Ks_ were measured using 1000 ms (I_Kr_) or 5000 ms-long (I_Ks_) test pulses between −30 mV and +50 mV (I_Kr_) or −20 to +50 mV (I_Ks_). The holding potential was −80 mV and during I_Kr_ measurements 500 ms long prepulse to −40 mV was applied in order to ensure the baseline region. The pulse frequency was 0.05 Hz (I_Kr_) or 0.1 Hz (I_Ks_). The decaying tail current at −40 mV after the test pulse was assessed as I_Kr_ or I_Ks_. The amplitudes of the I_Kr_ and I_Ks_ tail currents were determined as the difference between the peak tail current and the steady-state current level at −40 mV (baseline). When measuring I_Kr_, HMR-1556 (500 nM) was used to completely block I_Ks_, while dofetilide (0.1 µM) was added to the bath solution when studying I_Ks_. The top panels of Figure 5A and 5B show original I_Kr_ and I_Ks_ current traces in the absence and presence of 30 µM diclofenac, and indicate a significant blockade of I_Kr_ (at 20 mV test potential; from 57.7±5.5 pA to 36.2±2.3 pA, n = 5 cells/4 animals, p<0.05) and of I_Ks_ (at 20 mV test potential; from 229.6±15.0 pA to 126.5±10.5 pA, n = 6 cells/4 animals, p<0.05) by diclofenac. The corresponding bottom panels show the current-voltage relationships of I_Kr_ (Fig. 5A) and I_Ks_ (Fig. 5B) before and following superfusion with 30 µM diclofenac.

**Figure 6:**
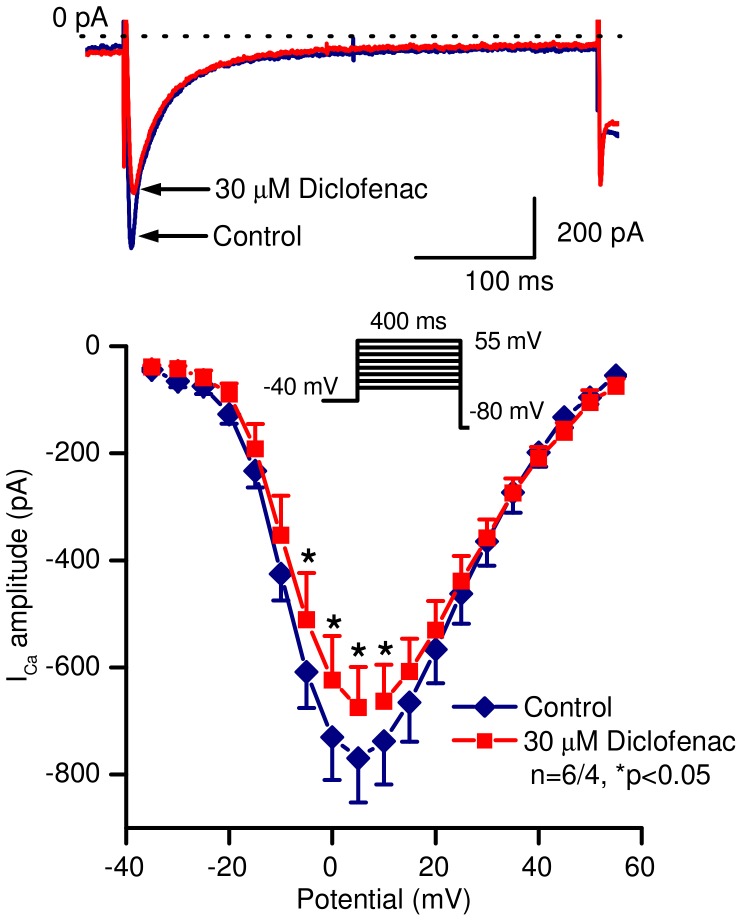
Effect of diclofenac on the L-type calcium current in canine ventricular myocytes. *Top* panel shows representative current traces, *bottom* panel represents current – voltage relationships under control conditions and in the presence of 30 µM diclofenac. Inset indicates the voltage protocol applied during measurements. Data are expressed as mean ± SEM, n = number of measurements/number of animals.

I_Ca_ was recorded in the presence 3 mM 4-aminopyridine in order to block I_to_. The current was evoked by 400 ms-long depolarizing test pulses to voltages between −35 to +55 mV. The holding potential was −80 mV and a 75 ms-long prepulse to −40 mV was applied in order to inactivate the sodium current. The pulse frequency was 0.2 Hz. The amplitude of I_Ca_ was defined as the difference between the peak inward current at the beginning and the current at the end of the pulse. Diclofenac (30 µM) slightly but statistically significantly decreased the amplitude of the current (at 0 mV test potential; from 730.5±79.8 pA to 623.2±82.4 pA, n = 6 cells/4 animals, p<0.05) as indicated on Figure 6.

## Discussion

The main results of the present study show that in the normal heart, diclofenac does not exert marked cardiac electrophysiological effects and does not enhance proarrhythmic risk, however, in hearts where repolarization reserve is attenuated, its moderate inhibition of I_Ks_ and I_Kr_ may lead to prolongation of ventricular repolarization and may also enhance proarrhythmic risk.

Our results indicate that diclofenac influences transmembrane ionic currents in canine ventricular myocytes, inhibiting I_Kr_, I_Ks_ and I_Ca_ currents but leaving I_to_ and I_K1_ unchanged. Only a slight action potential lengthening was induced in ventricular muscle preparations and in Purkinje fibers the drug shortened the action potential duration. The maximum upstroke velocity was decreased in both preparations by diclofenac. However, larger repolarization prolongation was observed when repolarization reserve was impaired by previous application of BaCl_2_. In anaesthetized rabbits, diclofenac did not prolong the QTc interval and did not induce TdP when administered alone. In contrast, when diclofenac was administered after the I_Kr_ blocker dofetilide it further increased the QTc interval and the incidence of TdP. In addition, dofetilide induced TdP in 20% of animals, however, when dofetilide was administered after diclofenac, the incidence of TdP was 62%.

For more than a decade, concerns about increased cardiovascular risks associated with NSAID drugs have been increasing, especially in patients with a history of cardiovascular disease [21], [22], [23]. The latest network meta-analysis of the cardiovascular safety of NSAID compounds involving more than 116 000 patients clearly suggested that NSAID drug administration was associated with elevated cardiovascular risk, and diclofenac was one of the drugs associated with the highest risk of cardiovascular death [12]. It is not clear from this meta-analysis, however, how the risk of serious ventricular arrhythmia induced sudden cardiac death was influenced by NSAIDs.

The present study focused on the effects of diclofenac on cardiac repolarization, action potential characteristics and on the main transmembrane ionic currents in ventricular muscle, since little is known about the direct cardiac electrophysiological effects and the possible proarrhythmic potency of this drug. Most of the information about the action of diclofenac on ionic currents arises from measurements in non-cardiac cells, activating the transient outward K^+^ current [24] and inhibiting sodium current [25], [26]. It was also observed that diclofenac enhanced KCNQ2/Q3 currents [27], others reported that the drug served as an activator of KCNQ4 and a blocker of KCNQ5 channels [28]. The only ionic current data obtained in ventricular cells were measured by Yarishkin *et al.* [29], who described inhibition of L-type Ca^2+^ current by the drug in neonatal rat ventricular myocytes.

Our results showed that diclofenac did not influence I_to_ and I_K1_ currents even at high concentration but decreased the amplitude of I_Kr_ and I_Ks_ currents in canine ventricular myocytes. In spite of the significant I_Kr_ blockade by the drug just a small but statistically significant action potential lengthening was detected following diclofenac administration in canine ventricular muscle. Some of our other observations may explain these seemingly conflicting results. Diclofenac significantly decreased the maximum upstroke velocity in canine ventricular muscle and also in Purkinje fibers indicating the Na^+^ channel blocking property of the drug. It is well established that the late or persistent component of the Na^+^ current contributes to the action potential plateau, which is most significant in Purkinje fiber [16]. In Purkinje fiber, diclofenac shortened the action potential duration most probably due to inhibition of the late Na^+^ current. Therefore, blockade of this current tends to limit the action potential prolongation resulting from the I_Kr_ inhibition by diclofenac. Indeed, a similar reduction of the action potential duration prolonging effect by additional I_Ca_ inhibition was demonstrated earlier in the case of the neuroleptic risperidone that blocks I_Kr_ [30]. Therefore, the slight decrease of L-type Ca^2+^ current by high concentration of diclofenac found in this study may also help to counteract the action potential lengthening effect of I_Kr_ blockade. The key role of I_Ks_ in ventricular repolarization was discussed in earlier works [7], [8], [31]. Full inhibition of I_Ks_ caused only a slight lengthening of repolarization in ventricular preparations, thus in normal canine ventricular muscle I_Ks_ plays a minor role in control of APD. This current, however, could provide an important means of limiting excessive APD lengthening when action potential duration is prolonged beyond normal by other mechanisms, thus contributing significantly to repolarization reserve [8]. Therefore, I_Ks_ blockade caused by diclofenac might only marginally influence action potential duration but attenuates repolarization reserve.

We characterized *in vitro* and *in vivo* electrophysiological effects of diclofenac: (i) the drug at high concentration inhibits both inward – depolarizing – and outward – repolarizing – transmembrane ionic currents in canine ventricular myocytes, (ii) resulting in only slight repolarization lengthening of ventricular muscle and shortening of the action potential in Purkinje fibers with *normal* repolarization reserve; (iii) diclofenac did not prolong the QT_c_ interval in anaesthetized rabbits. This implies that diclofenac may not augment spatial repolarization heterogeneity. However, in preparations with *impaired* repolarization reserve, much larger lengthening of the action potential duration was observed after application of the drug. Moreover, in our *in vivo* anaesthetized rabbit model when repolarization reserve was impaired, diclofenac further increased QT_c_ interval and the incidence of TdP while administered alone it did not induce any TdP.

According to the concept of repolarization reserve [1], [2], [32], normal repolarization is accomplished by multiple potassium channels providing a strong safety reserve for repolarization. Since inhibition or dysfunction of structural origin of one potassium channel does not necessarily lead to clinically manifest prolongation of repolarization, other potassium channels can take over the loss of function. However, a heart with impaired repolarization reserve is more vulnerable to arrhythmia development, since inhibition of another potassium channel by drugs may lead to significant inhomogenous repolarization prolongation and to serious cardiac dysrhythmias [3]. In the present study, the influence of diclofenac on action potential repolarization was also investigated after impairment of the repolarization reserve by adding 30 µM BaCl_2_, which partially blocks I_K1_ current. The drug induced a marked action potential lengthening, i.e. the APD lengthening effect of diclofenac was significantly larger in these conditions than in ventricular preparations with normal repolarization reserve.

Repolarization reserve can be reduced by several congenital or acquired pathophysiological conditions [3], as well as reversible cardiac hyperthrophy (athlete’s heart). Downregulation or dysfunction of the I_Ks_ current plays a critical role in the development of cardiac repolarization reserve impairment [33], [8]. In competitive athletes, slight impairment of repolarization reserve [34] does not result in a significant risk of arrhythmia but together with additional factors (increased sympathetic tone, seemingly harmless medications, doping agents, dietary constituents, hypokalemia, early and undiagnosed cardiomyopathy or other pathological anomalies), these hits on repolarization may add up and can cause repolarization abnormalities occasionally leading to sudden cardiac death (for a recent review see [5]). In this regard, we found higher beat-to-beat variability of the QT interval, a novel ECG parameter characterizing temporal instability of cardiac repolarization [35], in professional soccer players compared to their age-matched controls with no significant sports activities [36].

The applied concentration of diclofenac in the present study was somewhat higher than the reported therapeutic blood level, which is approximately 2–7 µM/L based on the data after 50 mg oral administration of diclofenac [37], [38]. Diclofenac strongly binds to plasma albumin [39], complicating the comparison of *in vitro* and *in vivo* data. In addition, substantial interpatient variabilities in unbound NSAID plasma concentrations and a poor correlation between concentration and therapeutic response to NSAIDs have been found [39]. It is well known that top athletes widely use NSAIDs, to treat their sports injuries frequently diclofenac, in larger doses [13] that can result in significantly higher plasma and tissue levels. In an *in vivo* animal study, application of diclofenac in therapeutic doses resulted in higher plasma concentration: a single 50 mg oral dose of diclofenac led to 6 µg/mL (approximately 20 µM/L) peak plasma concentration in Beagle dogs [40]. In clinical settings, diclofenac plasma levels can be increased by dietary constituents (e.g. grapefruit, pineapple juices) and co-administered medications interfering with its metabolism [41], [42], [43], [44], [45] or plasma protein binding [46].

### Study Limitations

BaCl_2_ was used as a tool to impair repolarization reserve in ventricular muscle preparations only, since in Purkinje fibers it was reasonable to expect that BaCl_2_ would have affected the slope of diastolic depolarization and would have influenced action potential measurements.

The actual plasma levels of diclofenac in anesthetized rabbits were not measured under the present experimental conditions, we relied on firm data from the literature to determine the dose and concentrations of diclofenac used in the experiments.

### Conclusions

*In the normal heart*, diclofenac at therapeutic and even at higher concentrations does not markedly influence ventricular repolarization and arrhythmia risk. However, high-dose treatment with the drug may enhance proarrhythmic risk in situations *that lead to reduced repolarization reserve*. Therefore, individuals taking diclofenac under proper medical control should not be concerned about proarrhythmic side effects, however, its administration may add to increased risk for serious arrhythmia development in persons associated with subsidiary risk factors including certain diseases or genetic defects that impair repolarization, as well as in individuals taking part in top competitive sports activities. Additional clinical studies are needed to elucidate whether diclofenac increases proarrhythmic risk in patients with congenital and/or acquired diseases and conditions associated with impaired repolarization reserve.
